# The carbon components in indoor and outdoor PM_2.5_ in winter of Tianjin

**DOI:** 10.1038/s41598-021-97530-x

**Published:** 2021-09-09

**Authors:** Baoqing Wang, Yinuo Li, Zhenzhen Tang, Ningning Cai

**Affiliations:** The State Environment Protection Key Laboratory of Urban Particulate Air Pollution Prevention, College of Environmental Science and Engineering, Nankai University, Tianjin, 300071 China

**Keywords:** Environmental sciences, Environmental chemistry

## Abstract

To study the carbon components in indoor and outdoor PM_2.5_, the samples of PM_2.5_ were collected from Nankai University in December 2015. The contents of eight carbon components were analyzed to use the thermo-optical reflection method. The results indicated that organic carbon (OC) mass concentration was 17.01, 19.48 and 18.92 µg/m^3^ in outdoor, dormitory and laboratory; elemental carbon (EC) mass concentration was 7.97, 3.56 and 3.53 µg/m^3^ in outdoor, dormitory and laboratory; and the total carbon aerosol was the proportion of more than 23% of PM_2.5_ samples. Lower wind speed and higher relative humidity were helpful to the accumulation of PM_2.5_. The ratio of OC/EC was > 2, and the SOC/OC ratio was > 30%, indicating that SOC was a crucial component indoors and outdoors. About 72% and 85% of the outdoor OC entering dormitory and laboratory environment, and about 59% and 71% of the outdoor EC entering dormitory and laboratory environment. Factor analysis of the eight carbon fractions indicated that the sources of OC and EC in outdoor, dormitory and laboratory is different.

## Introduction

In recent years, PM_2.5_ pollution events in northern China, such as in the Beijing-Tianjin-Hebei region^1^, have occurred frequently. The organic carbon (OC) and elemental carbon (EC) of carbon aerosols are crucial components in PM_2.5_. Due to the different environmental conditions and pollution sources in various regions, the total mass fraction of the carbon aerosols in PM_2.5_ ranges from 10 to 70% ^2–7^. The EC is mainly from the direct discharge of fossil fuels and biomass combustion, which is stable in the atmosphere. However, OC sources are relatively complex, which include both primary organic carbon (POC) and secondary organic carbon (SOC)^8,9^. OC and EC have an effect of the optical properties of the atmosphere and exert a substantial influence on the atmospheric visibility, climate change, and environmental quality^9,10^. Moreover, aerosol particles can easily enter human lungs, increasing the incidence of respiratory diseases and risk of cardiovascular diseases, which seriously threatens the human health^10–12^.

Research on the carbon aerosol in PM_2.5_ is popular in the field of international atmospheric aerosol^13,14^. Many cities, such as Seoul and Cheju (South Korea)^15^, Atlanta (United States)^16^, and Chiba (Japan)^17^, have conducted relevant studies to obtain the atmospheric particulate concentration level, seasonal characteristics, particle size distribution, and contribution sources of PM_2.5_ in various regions. Research on PM_2.5_ in China mainly focused on Shanghai^18,19^, Nanjing^20^, Guangzhou^21^, and Beijing-Tianjin-Hebei region^22–24^. The SOC formation and source characteristics of carbonaceous aerosols in PM_2.5_ samples were collected at the top of Mount Tai in the summer of 2015. The results indicating the fact that aqueous-phase reaction in droplets was an important pathway for SOC formation^25^. A comprehensive measurement of characteristics of carbonaceous aerosol (CA) and mass absorption cross-section (MAC) of elemental carbon (EC) in total suspended particles (TSP) collected from February 2015 to March 2017 in the southwest part of Karachi. Relatively lower OC/EC ratio (4.20 ± 2.50) compared with remote regions further indicated fossil fuel combustion as a primary source of CA^26^. The seasonal transport trends and potential source regions of carbonaceous species (OC, EC, SOC) of PM_10_ was evaluated over Darjeeling, an eastern Himalayas of India during August 2018–June 2019. The results indicated that biomass burning could be one of the major sources of carbonaceous aerosols in Darjeeling^27^. The spatio-temporal variation in EC, OC and SOC in PM_2.5_ fraction were performed in five sites of Indo-Gangetic plain. The change in concentration of OC was insignificant during summer. The OC/EC ratio (7–11) and OC/K^+^ ratio (10–52) during winter suggested the dominance of biomass burning from wood fuel and agricultural waste. EC was nearly constant at all sites suggesting the dominant presence of traffic-originated^28^. The carbonaceous fraction in PM_2.5_ from a tropical area in Malaysia, during January 2019 to March 2019. Secondary organic sources and primary sources emitted 46% and 54% of OC, respectively. The estimated char-EC was 10-fold higher than soot-EC, indicating that biomass burning and coal combustion were the predominant routes, whereas petrol or diesel engines were the less predominant generators^29^.

Indoor and outdoor concentrations of OC and EC in PM_2.5_ were analyzed for five buildings located near roadsides (an office and a classroom with mechanical ventilation (MV) and three residences with natural ventilation (NV)). The average I/O ratios of OC and EC were 1.02 and 0.80, respectively. The major source of indoor EC, OC and PM_2.5_ appears to be penetration of outdoor air ^30^. The indoor–outdoor characteristics of PM_2.5_ carbonaceous species in six residences were evaluated in Hong Kong during March and April 2004. The average I/O ratios of 24 h PM_2.5_, OC and EC were 1.4, 1.8, and 1.2, respectively. A simple model implied that about two-thirds of carbonaceous particles in indoor air are originated from outdoor sources^31^. Indoor and outdoor emission measurements to quantify the carbonaceous matters were performed from the real-world biomass burning in rural households. Fractions of fugitive emissions of the total reached as high as 44–48%. Fugitive emissions would result in very high peak concentrations of approximately tens of mg/m^3^^32^. Concurrent indoor–outdoor PM_2.5_ measurements were conducted at urban, suburban, and rural sites in Harbin, China. OC/EC and potassium ion to elemental carbon (K^+^/EC) ratios verified that biomass was an important source. The highest SOC/OC ratio was found at urban sites, up to 38.3% for indoors. SOC/OC ratios of indoor environments were higher, which is attributed to the conducive condition of forming the secondary pollutants during the heating period^33^. Studies have indicated that people spend 85%–90% of their lives indoors^34^. Few studies have focused on student dormitories and laboratories, which have distinct management and usage patterns. Unfavorable conditions in dormitory and laboratory environments can harm the health of the occupants.

Therefore, the PM_2.5_ samplings were conducted in outdoors and dormitory and laboratory in the Jinnan campus of Nankai University. The PM_2.5_ concentration, OC and EC correlation, SOC generation, and carbon pollution sources for outdoor, dormitory, and laboratory samples were analyzed and discussed. The results can provide reasonable suggestions for the prevention and control of atmospheric pollution.

## Materials and methods

### Sample collection

**Figure 1:**
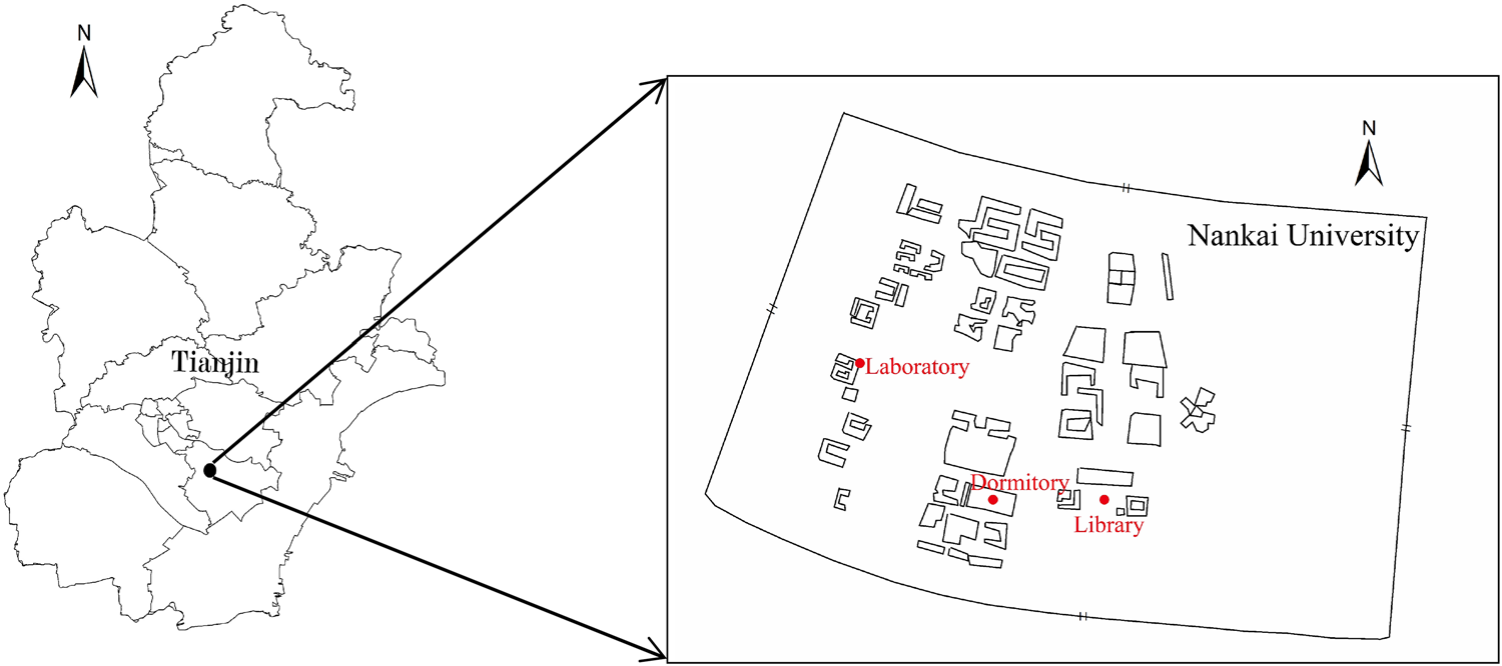
Sampling sites in this study (created by author).

The Jinnan campus of Nankai University was operational in September 2015. The outdoor sampling point was selected from Nankai University Library. The indoor sampling points were the laboratory and student dormitory. The sampling site was at an altitude of 30 m above ground level for indoor (laboratory and dormitory) and outdoor sampling (Library). The sampling point was 1.5 m above the floor for indoor and outdoor sampling. Sampling time is about 9 am-9 pm per day on December 3–21, 2015. The sampling sites in this study are shown in Fig. 1.

Before all the samples were measured, the parallel experiment of different instruments for different samplers indoor and outdoor were performed. The relative standard deviation of quality control of PM_2.5_, OC and EC concentration were less than 2.8%, 3.6% and 4.3%. For the collection of PM_2.5_ from the outdoor environment, a sampler (Wuhan Tianhong Company) with a flow rate of 100 L/min was used. The particle sampler equipped with a quartz filter was set at an outdoor monitoring point. The diameter of the filter was 90 mm. The flow rate of the sampler was calibrated before sampling to maintain the flow rate error within the acceptable range. For indoor PM_2.5_ sample collection, the LP-5 personal sampling pump (BUCK company, USA) and SKC personal exposed sampling cutting head (cut particle size: 2.5 μm) were used. A quartz filter with a diameter of 37 mm was used. The flow rate of the sampling pump was calibrated using the mini-buck soap film flow meter (BUCK company, USA).

Before sampling, the quartz filter was placed in a muffle furnace at 800 °C for 6 h to remove organic components^35^. Before weighing, the sample was placed in a constant temperature of 25 °C and relative humidity of 50% for 72 h. Subsequently, each sample was weighed twice to obtain an average value with an error of < 5 μg. After weighing^36^, the samples were preserved in a refrigerator (4 °C) for further analysis.

### Carbon analysis

The mass concentration of PM_2.5_ was obtained using the filter weighing method. The OC and EC were analyzed by using the thermal/optical carbon analyzer, and the analysis method was thermo-optical reflection (TOR)^12,37,38^. A 0.210-cm^2^ sample was obtained from the filter and sent to the thermo-optic analyzer. In a pure helium atmosphere, the temperature was increased to 120, 250, 450, and 550 ℃, and the particulate carbon on the filter was converted into CO_2_ to obtain four components of OC (OC1, OC2, OC3, and OC4). Then, the sample was gradually heated at 550, 700, and 800 °C in the helium atmosphere containing 2% (volume fraction) oxygen to obtain EC (EC1, EC2, and EC3). As the temperature increases in the inert helium, some of the OC forms optical pyrolized carbon (OPC) by the reflectance of 633 nm light from a He–Ne laser. The IMPROVE protocol defines OC as OC1 + OC2 + OC3 + OC4 + OPC and EC as EC1 + EC2 + EC3-OPC^39^.

### Element analysis

K, Zn, As, and Pb were used as tracer elements to analyze the carbon component sources of PM_2.5_. These elements are measured using inductively coupled plasma–mass spectroscopy (ICP–MS) (Agilent 7500a, Agilent Co. USA).

### Indoor-outdoor relationship

I/O represents the ratio of the indoor and outdoor concentration of particulate matter or of a compound. Researchs showed that simple linear correlation equation of indoor and outdoor concentration of particulate matter or of a compound can identify the contribution made by the two sources. The linear equation is used by many researchers^40^.

1 $$ C_{in} = F_{INF} + C_{out} + C_{ig} $$

where, $C_{in}$ refers to the particulate matter or compound concentration in indoor environment; $F_{INF}$ refers to the filtration factor, the fraction of outdoor particulate matter or compound that comes indoor environment; $C_{out}$ refers to the particulate matter or compound concentration in outdoor environment; $C_{ig}$ refers to the concentration of particle or compound generated from indoor sources.

### Meteorological data

Local meteorological data included temperature, relative humidity (RH), precipitation and wind speed were obtained from the website https://darksky.net/details/39.202,117.263. The meteorological data were collected each day during the sampling period.

## Results and discussion

### Concentration of PM_2.5_, OC, and EC

**Figure 2:**
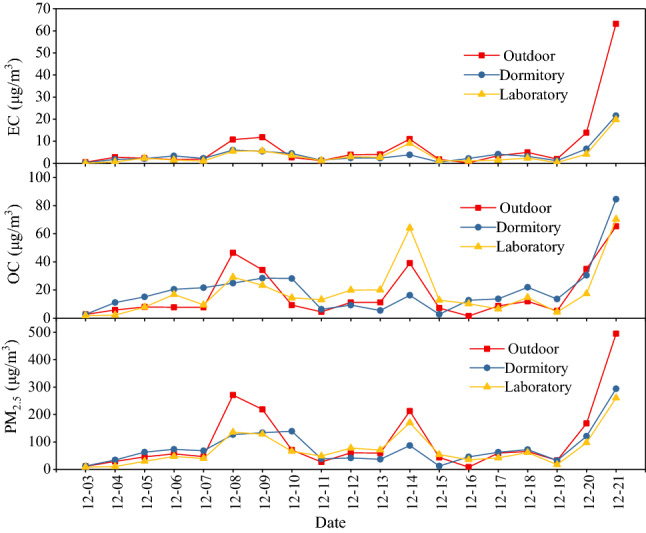
PM_2.5_, OC, and EC mass concentration in the outdoor, dormitory, and laboratory samples.

As shown in Fig. 2, the PM_2.5_ concentrations in outdoor, dormitory, and laboratory samples was in the range of approximately 8.5–494.41, 12.14–293.91, and 8.57–260.42 μg/m^3^, respectively. The OC and EC concentrations ranged from 2.71 to 65.31 μg/m^3^ and 0.48 to 63.16 μg/m^3^ for outdoor samples, 2.65 to 84.51 μg/m^3^ and 0.28 to 21.57 μg/m^3^ for dormitory samples, 1.80 to 70.39 μg/m^3^ and 0.07 to 19.77 μg/m^3^ for laboratory samples, respectively. The indoor PM_2.5_ and OC concentrations were considerably higher than those of the outdoor environment during the three days, which indicated that the indoor PM_2.5_ and OC are produced by indoor sources, such as personal activities and cleaning^21^. The concentration of PM_2.5_, OC, and EC increased on December 08, 14, and 21 mainly due to the heavy haze pollution, higher air relative humidity (93%, 82% and 84%), and lower wind speed (0.8 m/s, 1.6 m/s and 0.7 m/s).

The average outdoor, dormitory, and laboratory OC concentrations were 17.01, 19.48, and 18.92 μg/m^3^, respectively. The average OC concentration in indoor was higher than that in outdoor, and the OC concentrations in indoor and outdoor appeared as an asynchronous change in hazy weather. This may have caused by the pollutants accumulating indoors due to the indoor confinement and the low ventilation rate (windows and doors closed during in hazy weather, however windows and doors opened about 1–2 h during nonhazy weather). The average outdoor, dormitory, and laboratory EC concentrations were 7.57, 3.96, and 3.53 μg/m^3^, respectively. The indoor EC concentrations were lower than those of outdoor samples, which suggested that EC mainly came from primary outdoor sources such as incomplete coal burning and vehicle exhaust emission^38,39,41^. The outdoor, dormitory, and laboratory TC to PM_2.5_ ratios were 23.6%, 29.8%, and 30.5%, respectively, indicating that carbon was a crucial component of PM_2.5._

In this study, to evaluate the pollution levels of outdoor OC and EC in winter, the obtained carbon component concentration was compared with that of major cities at domestic and abroad. Table 1 indicates that the OC concentration level observed was lower than that observed in Tianjin (22.80 μg/m^3^)^24^, Beijing (21.91 μg/m^3^)^42^, Wuhan (23.30 μg/m^3^) ^43^, and Xining (19.27 μg/m^3^)^44^ and considerably higher than that observed in Xiamen (9.87 μg/m^3^)^45^, Shanghai (7.77 μg/m^3^) ^46^, and Taiwan (10.40 μg/m^3^)^8^. The EC concentration observed was lower than that observed in Wuhan (22.20 µg/m^3^)^43^ only, mainly because a large amount of coal was burned during winter, and the pollutants did not easily spread in temperature inversion. The OC and EC concentrations were considerably higher than those in Japan (3.75 µg/m^3^ and 1.63 µg/m^3^)^17^, Milan (14.00 µg/m^3^ and 1.60 µg/m^3^)^47^, and Greece (8.44 µg/m^3^ and 5.29 µg/m^3^)^48^, indicating relatively high carbon concentration in China. The carbon component concentration was high during winter in Tianjin, and the EC pollution was relatively serious. Therefore, it is necessary to control the primary carbon component emissions in Tianjin.

**Table 1:** OC/EC and SOC in PM_2.5_ in different cities.

Site	Date	PM_2.5_	OC	EC	OC/EC	SOC
This study	2015-12	104.22	17.01	7.57	2.25	9.18
Tianjin^23^	2014-01 ~ 2014-02	142.30	22.80	3.80	6.00	8.0
Xiamen^33^	2014-12 ~ 2015-01	74.38	9.87	1.87	5.28	3.50
Beijing^30^	2011-12 ~ 2012-01	90.69	21.91	5.03	4.47	–
Wuhan^31^	2009-12	137.00	23.30	22.20	1.05	6.2
Xining^32^	2014-11 ~ 2015-01	72.57	19.27	4.09	4.71	6.64
Shanghai^34^	2009-01 ~ 2009-02	88.30	7.77	1.33	5.84	2.49
Japan^16^	2013-12 ~ 2014-02	16.68	3.75	1.63	2.30	1.68
Milan^52^	2003-12	58.30	14.00	1.60	8.75	–
Taiwan^37^	1998-11 ~ 1999-04	68.00	10.40	4.00	2.60	4.20
Greece^36^	2012-02 ~ 2012-04	31.20	8.44	5.29	1.86	–

**Figure 3:**
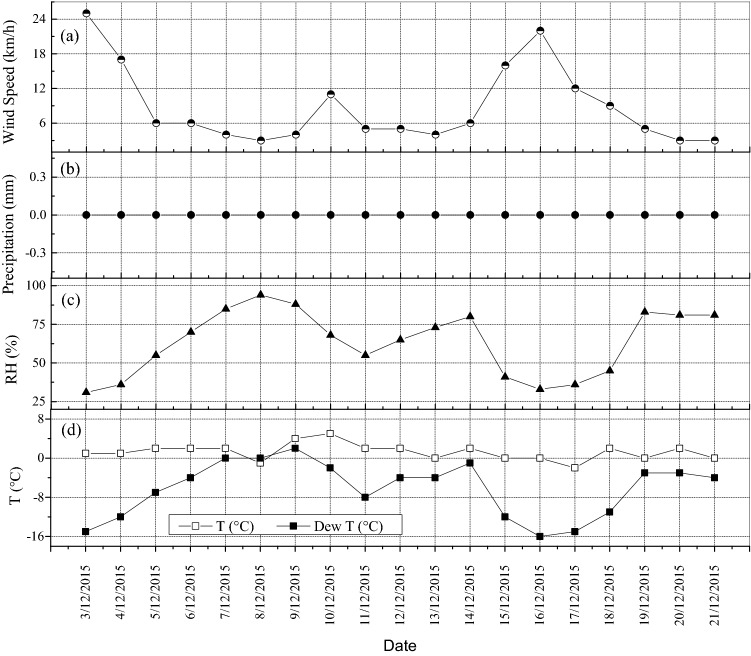
Meteorological conditions during the period of this study.

Meteorological conditions during the period of this study were shown in Fig. 3. Figure 3a–d indicated that the wind speed and relative humidity, wind speed and temperature are negative correlation. Lower wind speed and higher relative humidity were helpful to the accumulation of PM_2.5_. However, PM_2.5_ accelerated the diffusion with lower temperature and wind speed increasing. From the above analysis, the meteorological conditions including wind speed, relative humidity and temperature have important influence on the formation and diffusion of PM_2.5_.

### Indoor-outdoor relationship analysis

**Figure 4:**
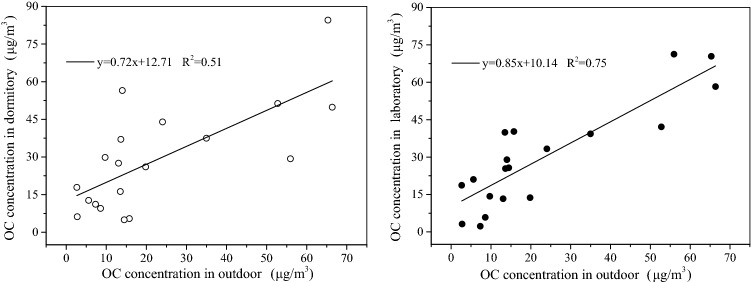
The regression analysis of OC concentration for dormitory and outdoor, laboratory and outdoor.

**Figure 5:**
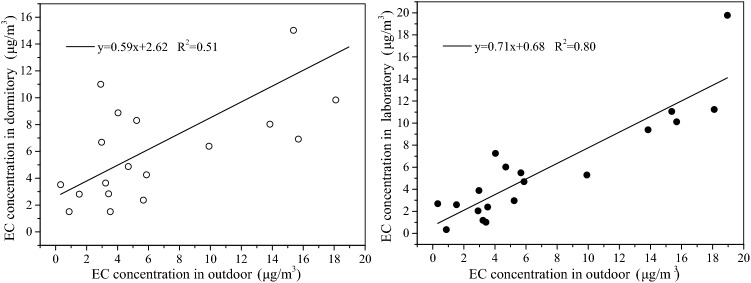
The regression analysis of EC concentration for dormitory and outdoor, laboratory and outdoor.

The regression analysis of OC concentration for dormitory and outdoor, laboratory and outdoor were shown in Fig. 4. The regression analysis of EC concentration for dormitory and outdoor, laboratory and outdoor were shown in Fig. 5.

According to Eq. (1), the filtration factors of the dormitory and outdoor, the laboratory and outdoor for OC were 0.72 and 0.85, which stated about 72% and 85% of the outdoor OC entering dormitory and laboratory environment, respectively. The filtration factors of the dormitory and outdoor, the laboratory and outdoor for EC were 0.59 and 0.71, which stated about 59% and 71% of the outdoor EC entering dormitory and laboratory environment, repectively.

### OC/EC ratios and SOC

SOC (secondary organic carbon) is a crucial source of carbon in PM_2.5_. Chow (2001)^39^ indicated that EC was relatively stable and can be used as a tracer of primary aerosol in the atmosphere^49^. The ratio of OC to EC was often used as the identification parameter for the existence of SOC. The existence of SOC was proven when OC/EC > 2. Table 2 showed that the average outdoor, dormitory, and laboratory OC/EC ratio was 2.25, 4.92, and 5.36, respectively, indicating that the proportion of SOC to OC was considerably large, and the indoor SOC pollution was more serious than the outdoor SOC pollution. The outdoor OC/EC ratio was lower than that in Xiamen (5.28)^45^, Beijing (4.47)^42^, Xining (4.71)^44^, Shanghai (5.84)^46^, Milan (8.75)^47^, Japan (2.30)^17^, and Taiwan (2.60)^8^ and was higher than that in Wuhan (1.05)^43^ and Greece (1.86)^48^, which indicated that SOC pollution was relatively serious in China.

**Table 2:** Mass concentration of indoor and outdoor PM_2.5_, OC, EC, and SOC.

Site	PM_2.5_ (µg/m^3^)	OC (µg/m^3^)	EC (µg/m^3^)	OC/PM_2.5_ (%)	EC/PM_2.5_ (%)	OC/EC	SOC (µg/m^3^)	SOC/OC (%)
Outdoor	104.22	17.01	7.57	16.3	7.3	2.25	9.21	54.14
Dormitory	78.64	19.48	3.96	24.8	5.0	4.92	10.41	53.47
Laboratory	73.69	18.92	3.53	25.7	4.8	5.36	7.15	37.77

To quantitatively describe SOC, Turpin (1995) proposed the following empirical equation:

2 $$ SOC = OC_{tot} - EC \times \left( {\frac{OC}{{EC}}} \right)_{\min } $$

where, OC_tot_ stands for the total organic carbon, EC stands for the elemental carbon, and (OC/EC)_min_ stands for the minimum of OC/EC.

The SOC concentrations are shown in Table 1. The outdoor SOC concentration was 9.21 μg/m^3^, accounting for 54.14% of OC. The SOC concentrations in the dormitory and laboratory were 10.41 and 7.15 μg/m^3^, accounting for 53.47% and 37.77% of OC, respectively. These results indicated that SOC in this study was relatively active. The SOC concentration in the dormitory was higher than the outdoor and laboratory SOC concentrations, most likely because volatile organic compounds emitted from some sources^8,50^, the temperature in the dormitory was higher than that outdoors and in the laboratory during heating, which generated SOC^9,51^. Meanwhile, poor indoor air circulation contributes to the high concentration of SOC^30^.

### Correlations Between OC and EC

Turpin (1995)^52^ found that the correlation between OC and EC concentration can be used to qualitatively analyze the source of atmospheric carbon aerosols. The correlation between OC and EC concentration can be expressed by a linear regression equation,

3 $$ {\text{Y }} = {\text{ AX }} + {\text{ B}} $$

where, Y stands for the concentration of OC, X stands for the concentration of EC, intercept B is the OC generated by the chemical reaction of organic matter in the atmosphere, and slope A reflects a change in the emission characteristics of pollution sources in a region.

**Figure 6:**
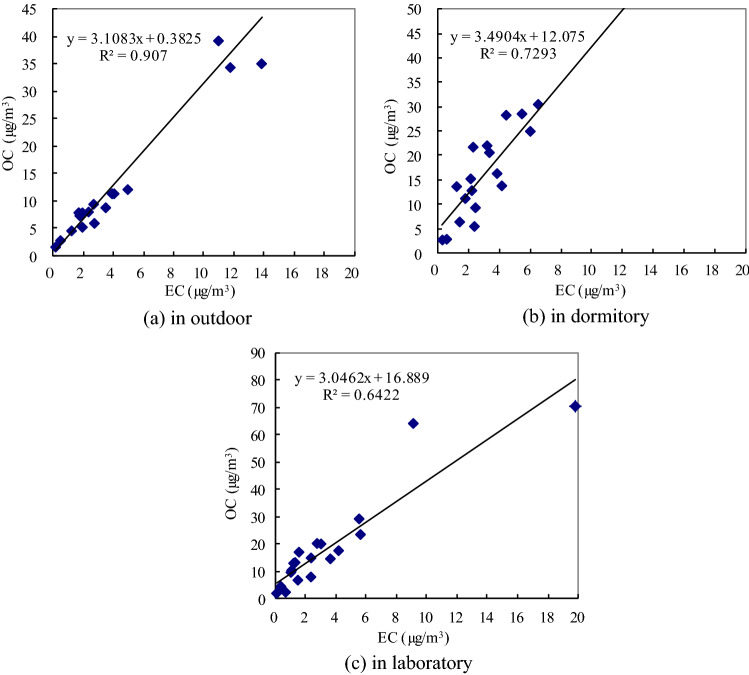
Correlation between OC and EC in indoor and outdoor.

Figure 6 showed that the correlation between OC and EC (0.9070) in outdoor samples was the strongest, indicating that OC and EC may have similar sources. Related research has shown that coal combustion and vehicle exhaust emissions accounted for approximately 83% of the annual emissions of OC and EC in China ^9,51,53–55^; therefore, coal burning and vehicle exhaust emission may be the main sources of pollution. The correlation between OC and EC in dormitory and laboratory samples were 0.7293 and 0.6422, which were lower than that in outdoor samples, mainly due to photochemical reactions and indoor OC emission sources.

### Source analysis

#### Tracer elements

K, Zn, As, and Pb were used as tracer elements to analyze the carbon component sources of PM_2.5_. These elements are measured using inductively coupled plasma–mass spectroscopy (ICP–MS) (Agilent 7500a, Agilent Co. USA). K indicated biomass burning and waste combustion. Zn and Pb were tracer elements of vehicle emissions. As indicated the contribution of coal combustion sources ^50,56^. The correlation between OC, EC, and tracer elements is presented in Table 3.

**Table 3:** Correlation between OC, EC, and tracer elements.

Site	Project	K	Zn	As	Pb
Outdoor	OC	0.971**	0.955**	0.909**	0.954**
	EC	0.864**	0.911 **	0.782**	0.918**
Dormitory	OC	0.700**	0.890**	0.645**	0.929**
	EC	0.632**	0.946**	0.591**	0.957**
Laboratory	OC	0.794**	0.799**	0.508*	0.753**
	EC	0.847**	0.864**	0.497*	0.862**

**Extremely significant correlation, *P* < 0.01; *Significant correlation, *P* < 0.05.

As shown in Table 3, the correlations of OC–K was highest outdoors, followed by OC-Zn and OC-Pb, which indicated OC outdoor was mainly from biomass burning and vehicle emission. For EC outdoor was mainly from vehicle emission. The correlations of OC–Zn and OC–Pb were the highest in the dormitory, which showed that OC in the dormitory was mainly from the vehicle exhaust, for EC in the dormitory was mainly from the vehicle exhaust. The correlations of OC–Zn, OC-K and OC–Pb were the highest in the laboratory, which showed that OC in the laboratory was mainly from the vehicle exhaust and biomass burning, for EC in the laboratory was mainly from the vehicle exhaust and biomass burning. The recent study has suggested that residential coal burning in the rural areas is an important source of organic matters in north China in winter^57^.

#### Factor analysis

The principle of TOR analysis of carbon aerosol components was the separation of eight carbon components from each sample by using different temperature gradients. Cao et al.^58^ observed that OC1 and OPC (optical pyrolized carbon) were abundantly produced during biomass combustion; OC2, OC3, OC4, and EC1 were abundantly produced during coal combustion and vehicle exhaust; EC2 and EC3 were abundantly produced during diesel vehicle exhaust. Therefore, the abundance distribution of eight carbon components can be used to analyze the source of carbon aerosol^59^.

**Figure 7:**
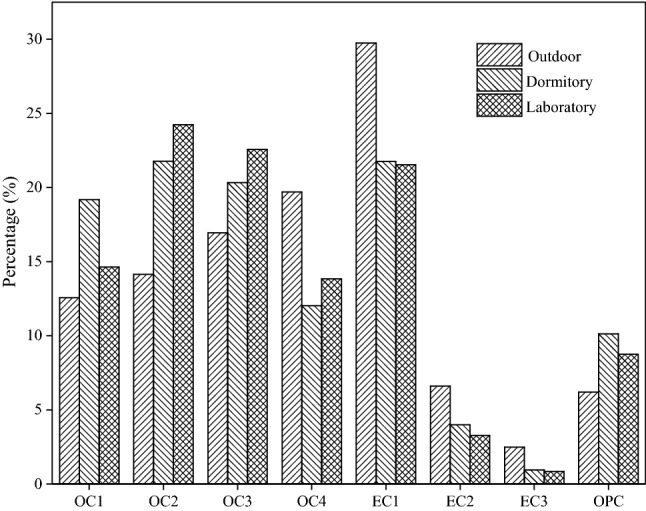
Ratio of eight carbon components to total carbon indoor and outdoor.

Figure 7 illustrates the abundance distribution of the carbon components observed in outdoor, dormitory, and laboratory samples during the sampling period. The results revealed higher abundance of EC1, OC2, OC3, OC4 and OC1 indoor and outdoor.

To quantify the main sources of carbon pollution in indoor and outdoor PM_2.5_, the eight carbon components was evaluated by factor analysis. The factor analysis for the carbon component in indoor and outdoor samples are presented in Table 4. The outdoor high load components for factor 1 were EC1 (0.926), OC3 (0.851), OC1 (0.833) and OC2 (0.832), which accounted for 74.61% of the carbon component and mainly represented OC was from coal combustion, gasoline vehicle exhaust emissions and biomass burning, EC was from coal combustion and gasoline vehicle exhaust emissions. The high load components in the dormitory for factor 1 were OPC (0.839) and EC2 (0.752) which accounted for 94.65% of the carbon component and represented OC was from biomass combustion and EC was from diesel vehicle exhaust emissions. The high load components in laboratory for factor 1 were OPC (0.987), EC2 (0.984), OC4 (0.982), EC1 (0.979), OC3 (0.937) and OC2 (0.909) which accounted for 84.76% of the carbon components and represented OC was from biomass burning, coal burning and gasoline vehicle exhaust emissions, EC was from coal combustion and vehicle exhaust emissions. The analysis using element tracers and factor analysis revealed that the sources of indoor and outdoor carbon components in this study were similar.

**Table 4:** Factor analysis of the carbon component in indoor and outdoor samples.

	Outdoor		Dormitory		Laboratory	
	Factor 1	Factor 2	Factor 1	Factor 2	Factor 1	Factor 2
OC1	0.833	0.356	0.527	0.479	0.853	0.127
OC2	0.832	0.461	0.678	0.525	0.909	0.193
OC3	0.851	0.465	0.748	0.487	0.937	0.244
OC4	0.554	0.802	0.606	0.685	0.982	0.095
EC1	0.926	0.221	0.711	0.583	0.979	0.166
EC2	0.414	0.716	0.752	0.437	0.984	0.030
EC3	0.394	0.268	0.403	0.852	0.114	0.993
OPC	0.154	0.119	0.839	0.424	0.987	0.083
Variance %	74.61	15.12	94.65	3.21	84.76	12.00

## Conclusion

In this study, the carbon components in indoor and outdoor PM_2.5_ in winter of Tianjin were investigated.

The mass concentration of OC from outdoor, dormitory, and laboratory samples were 17.01, 19.48, and 18.92 µg/m^3^, the mass concentrations of EC were 7.97, 3.56, and 3.53 μg/m^3^, and the ratios of TC to PM_2.5_ were 23.6%, 29.8%, 30.5%, respectively. This result indicated that carbonaceous aerosol was a crucial component in PM_2.5_, and the indoor pollution source certainly influenced the concentration of OC. Compared with major cities in China and abroad, the concentration of carbon components in this study was higher during winter; therefore, the control of carbon component emissions should be strengthened. Lower wind speed and higher relative humidity were helpful to the accumulation of PM_2.5_. However, PM_2.5_ accelerated the diffusion with lower temperature and wind speed increasing.

The filtration factors of the dormitory and outdoor, the laboratory and outdoor for OC were 0.72 and 0.85. The filtration factors of the dormitory and outdoor, the laboratory and outdoor for EC were 0.59 and 0.71. The average OC/EC for outdoor, dormitory, and laboratory samples were 3.58, 5.78, and 7.84, respectively. Moreover, the SOC/OC for outdoor, dormitory, and laboratory samples were 54.14%, 53.47%, and 37.77%, respectively. This finding indicated that SOC was actively produced in winter, and attention should be paid to SOC pollution.

The correlation coefficients of OC and EC for outdoor, dormitory, and laboratory samples were 0.9070, 0.7293, and 0.6422, respectively. The correlation between OC and EC in outdoor samples was higher than that in dormitory and laboratory samples, which indicated similarity between the pollution sources of OC and EC in outdoor samples. The elemental tracers and factor analysis method were used to analyze the quantitative contribution of carbon pollution in PM_2.5_. The results showed that the carbon aerosol indoors and outdoors was mainly from coal burning, vehicle exhaust, and biomass burning.

## Data Availability

The datasets generated during or analysed during the current study are available from the corresponding author on reasonable request.
