# Author Correction: The carbon components in indoor and outdoor PM_2.5_ in winter of Tianjin

**DOI:** 10.1038/s41598-021-98824-w

**Published:** 2021-09-21

**Authors:** Baoqing Wang, Yinuo Li, Zhenzhen Tang, Ningning Cai

**Affiliations:** The State Environment Protection Key Laboratory of Urban Particulate Air Pollution Prevention, College of Environmental Science and Engineering, Nankai University, Tianjin, 300071 China

Correction to: *Scientific Reports* 10.1038/s41598-021-97530-x, published online 09 September 2021

The original version of this Article omitted the Acknowledgements section. The Acknowledgements section now reads:

“This study was supported by National Natural Science Foundation of China (21777076)."

The original Article has been corrected.

